# Development of Bleeding Artificial Intelligence Detector (BLAIR) System for Robotic Radical Prostatectomy

**DOI:** 10.3390/jcm12237355

**Published:** 2023-11-28

**Authors:** Enrico Checcucci, Pietro Piazzolla, Giorgia Marullo, Chiara Innocente, Federico Salerno, Luca Ulrich, Sandro Moos, Alberto Quarà, Gabriele Volpi, Daniele Amparore, Federico Piramide, Alexandru Turcan, Valentina Garzena, Davide Garino, Sabrina De Cillis, Michele Sica, Paolo Verri, Alberto Piana, Lorenzo Castellino, Stefano Alba, Michele Di Dio, Cristian Fiori, Eugenio Alladio, Enrico Vezzetti, Francesco Porpiglia

**Affiliations:** 1 Department of Surgery, Candiolo Cancer Institute, FPO-IRCCS, 10060 Turin, Italy; volpi_gabriele@yahoo.it; 2 Department of Mechanical Engineering, Politecnico di Milano, 20156 Milano, Italy; pietro.piazzolla@polimi.it; 3 Department of Management, Production, and Design Engineering, Polytechnic University of Turin, 10129 Turin, Italy; giorgia.marullo@polito.it (G.M.); chiara.innocente@polito.it (C.I.); federico.salerno@polito.it (F.S.); luca.ulrich@polito.it (L.U.); sandro.moos@polito.it (S.M.); enrico.vezzetti@polito.it (E.V.); 4 Department of Oncology, Division of Urology, University of Turin, San Luigi Gonzaga Hospital, 10043 Orbassano, Italy; quara.alberto@gmail.com (A.Q.); danieleamparore@hotmail.it (D.A.); federico.piramide@gmail.com (F.P.); alexandru.turcan@edu.unito.it (A.T.); valentina.garzena@unito.it (V.G.); davide.garino@unito.it (D.G.); sabrinatitti.decillis@gmail.com (S.D.C.); michele.sica1991@gmail.com (M.S.); paoloverri05@gmail.com (P.V.); cristian_fiori@icloud.com (C.F.); porpiglia@libero.it (F.P.); 5 Romolo Hospital, Rocca di Neto (KR), 88821 Rocca di Neto, Italy; alb.piana@gmail.com (A.P.); stefanoalba78@gmail.com (S.A.); 6 Department of Chemistry, University of Turin, 10124 Torino, Italy; lorenzo.castellino@unito.it (L.C.); eugenio.alladio@unito.it (E.A.); 7 Antidoping Center “A. Bertinaria”, 10060 Turin, Italy; 8 Division of Urology, Department of Surgery, SS Annunziata Hospital, 87100 Cosenza, Italy; micheledidio@yahoo.it

**Keywords:** prostate cancer, artificial intelligence, complications, robotics

## Abstract

Background: Addressing intraoperative bleeding remains a significant challenge in the field of robotic surgery. This research endeavors to pioneer a groundbreaking solution utilizing convolutional neural networks (CNNs). The objective is to establish a system capable of forecasting instances of intraoperative bleeding during robot-assisted radical prostatectomy (RARP) and promptly notify the surgeon about bleeding risks. Methods: To achieve this, a multi-task learning (MTL) CNN was introduced, leveraging a modified version of the U-Net architecture. The aim was to categorize video input as either “absence of blood accumulation” (0) or “presence of blood accumulation” (1). To facilitate seamless interaction with the neural networks, the Bleeding Artificial Intelligence-based Detector (BLAIR) software was created using the Python Keras API and built upon the PyQT framework. A subsequent clinical assessment of BLAIR’s efficacy was performed, comparing its bleeding identification performance against that of a urologist. Various perioperative variables were also gathered. For optimal MTL-CNN training parameterization, a multi-task loss function was adopted to enhance the accuracy of event detection by taking advantage of surgical tools’ semantic segmentation. Additionally, the Multiple Correspondence Analysis (MCA) approach was employed to assess software performance. Results: The MTL-CNN demonstrated a remarkable event recognition accuracy of 90.63%. When evaluating BLAIR’s predictive ability and its capacity to pre-warn surgeons of potential bleeding incidents, the density plot highlighted a striking similarity between BLAIR and human assessments. In fact, BLAIR exhibited a faster response. Notably, the MCA analysis revealed no discernible distinction between the software and human performance in accurately identifying instances of bleeding. Conclusion: The BLAIR software proved its competence by achieving over 90% accuracy in predicting bleeding events during RARP. This accomplishment underscores the potential of AI to assist surgeons during interventions. This study exemplifies the positive impact AI applications can have on surgical procedures.

## 1. Introduction

Prostate cancer (PCa) is the first urological malignancy in men, and among the local treatment options, radical prostatectomy still remains the preferred one. In the last decades, the advent of robotics has led to a groundbreaking shift from open or laparoscopic surgery to robot-assisted radical prostatectomy (RARP), thanks to the well-denoted advantages for the surgeons and potential benefits for the patients [1]. This increasing number of RARPs around the world is related to an even higher number of young surgeons who are faced with this surgical approach; however, as recently reported, 33% of neo-urologists in the US are not able to perform robotic procedures at the end of their residency, and it could potentially lead to the occurrence of future intraoperative adverse events [2]. With the introduction of a standardized surgical training program [3,4], the field has witnessed the emergence of new technologies as significant contributors.

Recently, Artificial Intelligence systems were introduced in surgical scenarios with the aim of estimating surgical outcomes. The analysis of large amounts of patients’ information can permit the creation of algorithms that are able to predict surgical outcomes, especially in robotics.

One of the major challenges in laparoscopic/robotic surgery is dealing with intraoperative bleeding, which accounts for 23% of all adverse events. Recent efforts have focused on expediting the identification of bleeding during endoscopic procedures. Techniques utilizing RGB space parameters [5] or employing color features to categorize pixels as “blood” or “non-blood” have been explored. These methods leverage machine learning, specifically the Support Vector Machine (SVM), to process and classify information [6]. In the realm of deep learning, convolutional neural networks (CNNs) have proven to be a suitable option for automatically extracting features [7] and detecting adverse events [8]. CNNs have been employed to segment bleeding sources and present them to surgeons, classify images as bleeding or non-bleeding, and locate, recognize, and track bleeding points in real-time [9]. In this sense, multi-task learning (MTL) is a promising strategy within the realm of neural networks. Indeed, MTL allows for efficient simultaneous learning of multiple tasks, resembling the learning process of humans [10]. This is an I.D.E.A.L. (Idea, Development, Exploration, Assessment, Long-term) study 0-1. The research question is to develop an AI system able to predict the occurrence of intraoperative bleeding during robot-assisted radical prostatectomy (RARP) and alert the surgeon about the risk of bleeding. Our hypothesis is that our novel system, based on MTL-CNNs, can achieve this result.

## 2. Materials and Methods

Herein, we present a study with a preclinical stage (I.D.E.A.L. 0) dealing with the evaluation of our specifically developed software in a training phase. Then, our proof of concept was tested on human pre-recorded videos (I.D.E.A.L. 1), demonstrating how it works in a real-time video streaming setting.

### 2.1. MTL-CNN Development

**Figure 1:**
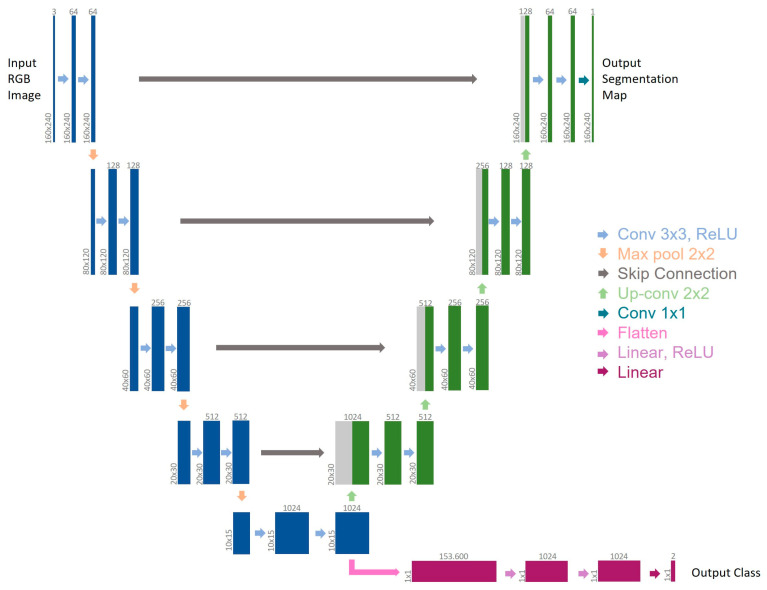
Architecture of the employed CNN. Multi-channel feature maps are represented by each box. The box top displays the number of channels, while its lower left border displays the x-y dimensions. The backbone is represented by blue boxes, the first branch for semantic segmentation is shown by green boxes, copied feature maps are indicated by gray boxes, and the second branch for event detection is indicated by purple boxes. The various operations are shown by the arrows.

Aiming to identify the principal actor in the action that strengthens and recognizes the action itself [11,12], an MTL-CNN [13], as an architecture that can simultaneously learn many tasks, was introduced in the current study. More specifically, the model could jointly execute semantic segmentation and event detection, namely bleeding recognition, during laparoscopic/robotic surgery. A new architecture was put into place with a dataset that had been correctly manually classified to achieve this goal. The architecture comprised a global feature extractor made up of convolutional layers shared by all tasks, followed by a separate branch for each output [14]. Each weight is trained to minimize multiple loss functions simultaneously due to the pooling of the weights for various tasks. The U-Net architecture [15] has been modified to create the MTL-CNN architecture, which is shown in Figure 1. The U-Net is a model that is frequently used in the literature because of the way it is built, which enables detailed analysis of medical data. All tasks share the contracting path (left side), which represents global feature extraction. In contrast to U-Net’s initial architecture, the bottleneck of the feature extractor results in two independent output branches, each of which handles a different task. Semantic segmentation is carried out via the first branch (right–top side), which is adapted from the expanded path of U-Net. The second branch, coupled with the encoder output as well, approaches event detection as a classification problem using the U-Net encoder as its starting point. Two classes might show up in the output in this case: 0 for “no blood accumulation” and 1 for “blood accumulation.”

### 2.2. BLeeding Artificial Intelligence DetectoR (BLAIR) Software Implementation

In this section, we present in detail the software application that leverages the neural network described in [11], called “Core NN,” in the following and is used for the clinical validation focus of this paper. The BLeeding Artificial Intelligence-based DetectoR, or BLAIR for short, has been developed in Python for rapid interfacing with the neural networks through the Keras API. From an architectural perspective, BLAIR exploits PyQT queues and treads to handle multiple tasks simultaneously. In particular, BLAIR receives as input the video stream from the DaVinci robot, as recorded or during a live surgical procedure, feeds the video frames to the Core NN, and provides the final user with insights concerning the likelihood that bleeding is about to occur in the next few instants based on the Core NN output. We base this approach on the Core NN validation tests that can be found in the same [11] paper. Since keeping track of surgical tool movement was not essential for the application’s main goal, we narrowed our attention for this purpose to the branch of the MTL-CNN that performs event detection. Therefore, the semantic segmentation branch was employed to enhance bleeding recognition performance during convolutional neural network internal calculation.

**Figure 2:**
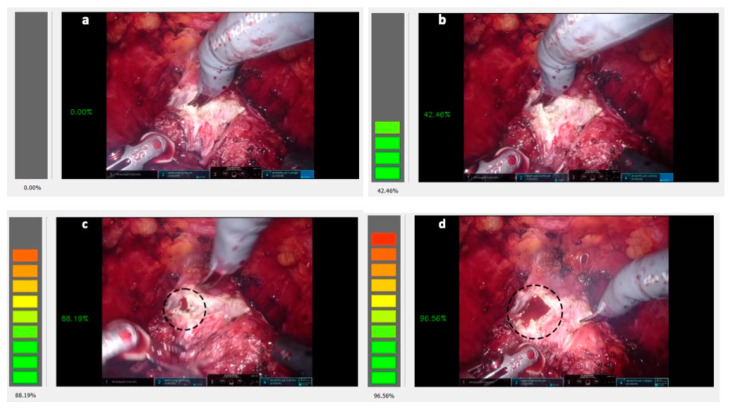
The BLAIR application interface. (**a**) No bleeding prediction by the Core NN. When repeated prediction with a small confidence percentage occurs in a time frame, the alert level in the left bar increases (**b**) up to the point when the bleeding event occurs, as it can be seen in the dotted circle (**c**). The gravity of the event does not change substantially the alert level (**d**).

The BLAIR interface is very straightforward, as can be seen from Figure 2a. Its main window shows the endoscope frame that has been fed to the Core NN, along with a warning bar whose color and height change accordingly, with a higher probability of imminent bleeding. The purpose of the bar is to be easily visible to the surgeon during the procedure when the BLAIR interface is shown through the TilePro feature of the DaVinci console.

Since the signal of the Core NN output is highly perturbed, the main innovative contribution of this paper with respect to [11] is the results obtained with the application of a low-pass filter (LPF) to reduce this signal noise and have a stabler output. When using an LPF, only signals below a defined cutoff frequency are allowed to pass it, while signals above the cutoff are attenuated. We used a Butterworth filter with a cutoff frequency of 5 Hz, which created a 20 ms delay that we considered acceptable in this application.

During the development and testing stages of the BLAIR application, we noticed that the Core NN output signal filtered with our custom-designed LPF in case of repeated detected possible bleeding occurrences, even if with a small confidence percentage but repeating over the course of a time frame of approximately 2100–2300 ms (Figure 2b), tends to precede a more noticeable bleeding event (Figure 2c). This feature has been integrated into the application design so that the filtered output signal can be used to alert the surgeon to an approaching bleeding event in a way that can be thought of as analogous to the functioning of a parking sensor for cars.

The BLAIR application is currently unable to distinguish the gravity of the bleeding; thus, a small or more significant event is detected with the same warning level (compare Figure 2c and Figure 2d). In future improvements, this limitation will be addressed, as will the introduction of precise information about the location of the organ of the probable bleeding event.

### 2.3. Clinical Evaluation of BLAIR Software Performances

With the aim of evaluating the real-time clinical performances of our specifically developed BLAIR software, the pre-recorded videos of 10 patients who underwent robot-assisted radical prostatectomy (RARP) plus extended lymph adenectomy at our center in 2023 were extracted. All the videos were reviewed by a dedicated urologist (A.Q.). At first, all videos were watched on standard video player software; every occurrence of bleeding was recorded on a dedicated database with its specific time of appearance.

Then, all the videos were launched on the BLAIR application, and again, we noted every bleeding occurrence. Significant bleeding on BLAIR software was defined with a threshold between 93 and 95%, according to our previously published experience [16].

The occurrence of the bleeding was registered in a dichotomic fashion (0: no bleeding; 1: bleeding).

### 2.4. Data Collection and Statistical Analysis

Preoperative and perioperative data were collected, especially in terms of estimated blood losses (EBL) and intraoperative complications.

The concordance of the identification of bleeding events between humans and AI, named “outcomes,” was registered; furthermore, the timing of the occurrence was noted.

Regarding the MTL-CNN training parameter optimization, a multi-task loss was adopted:

loss = seg_loss + cls_loss

where seg_lossis is a binary cross-entropy loss function and cls_loss is a cross-entropy loss function.

The following formula was used to determine the event detection branch accuracy:

Classification Accuracy = (#correct_predictions)/(#samples)

where #correct_predictions is the number of images that have been correctly classified, and #samples is the total number of test images.

Furthermore, to evaluate software performance, we applied the Multiple Correspondence Analysis (MCA) approach, which first provides a plot that helps identify variables that are most correlated with each dimension computed by MCA modeling. The squared correlations between variables and the dimensions are used as coordinates. MCA is a dimensionality reduction technique used for the exploration and visualization of categorical data. It is an extension of Correspondence Analysis (CA) designed to handle datasets with multiple categorical variables. MCA transforms the original high-dimensional categorical data into a lower-dimensional space, where relationships between categories and variables are preserved [17]. The technique identifies underlying patterns, associations, and dependencies among categorical variables, aiding in the interpretation and understanding of complex datasets. MCA is particularly useful when dealing with large contingency tables and complex categorical dataset structures commonly encountered in the social sciences, marketing, and survey data analysis. By providing a simplified representation of the data, MCA facilitates data exploration and visualization, enabling researchers to gain valuable insights and make informed decisions based on the patterns and relationships discovered within the categorical dataset. In this context, MCA was employed for exploratory purposes to evaluate whether the occurrence of true positive, false negative, and false positive results was significant when using AI or not.

## 3. Results

### 3.1. MTL-CNN Training Phase Findings

The findings of the MTL-CNN’s training phase are reported in this section.

**Figure 3:**
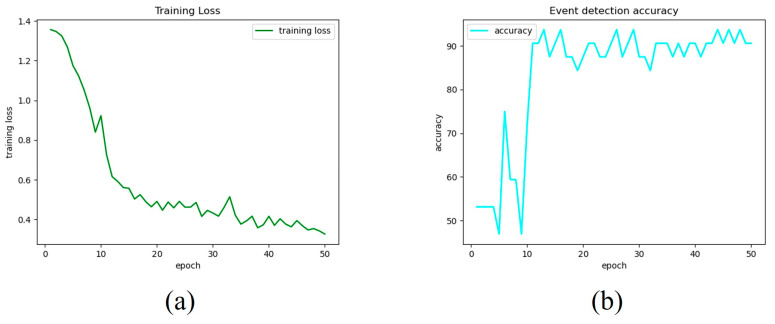
Training and validation metrics are trends. Training loss (**a**) and validation accuracy for the event detection branch (**b**).

Both images and videos were used to evaluate the multi-task CNN. Figure 3a,b demonstrates the trend of the training loss and event detection accuracy for each epoch. The final model was then chosen for epoch 30, which was determined by experimental tests on images and videos to be the best tradeoff between the two branches of the network.

Regarding the image tests, the MTL-CNN was able to recognize events with an accuracy of 90.63% without the use of any pre- or postprocessing techniques. Additionally, the accuracy for each class was reported, yielding accuracy for the classes “no blood accumulation” and “blood accumulation” of 86.67% and 94.12%, respectively.

### 3.2. Clinical Evaluation of BLAIR Software Performances

**Figure 4:**
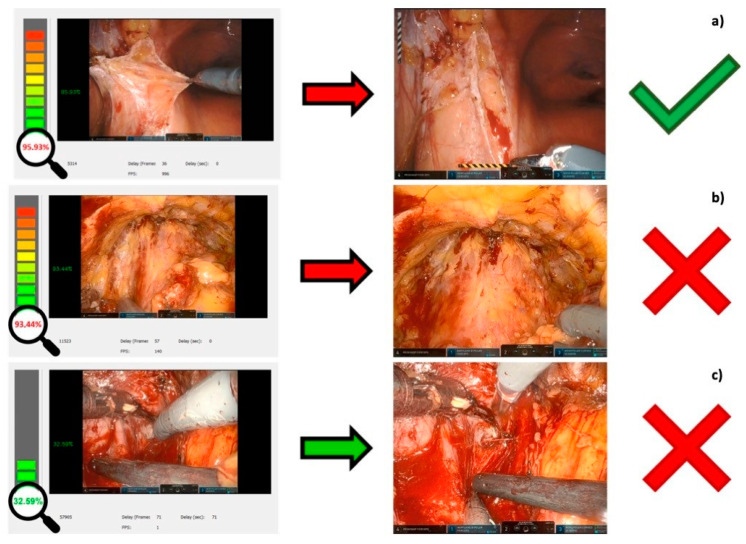
Examples of BLAIR software performance evaluations. (**a**) The software correctly predicts the bleeding (true positive); (**b**) the software wrongly predicts a bleeding that did not occur (false positive); (**c**) the software fails to predict a bleeding (false negative).

Clinical perioperative variables of the 10 patients whose videos have been reviewed are reported in Table 1, and an example of data recording for software evaluation is shown in Figure 4.

**Figure 5:**
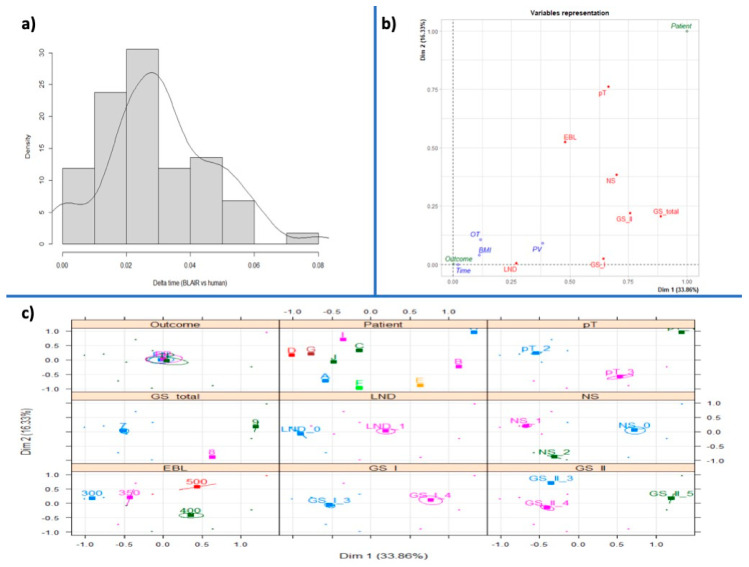
Clinical evaluation of BLAIR software performance. (**a**) Density plot of the delta time (BLAIR vs. human) in terms of seconds. The *x*-axis represents the difference in time between the BLAIR and clinical (human) measurements, and the *y*-axis represents the density of data points in that range. It reports a histogram of the collected data, together with a KDE (Kernel Density Estimation) plot, i.e., a smoothed representation of the data’s probability density function. (**b**) Plot of the MCA dimension. (**c**) Plot of the MCA dimensions in terms of the individuals under analysis.

The BLAIR software can predict and alert the surgeon to a potential bleeding occurrence in advance, as shown in the density plot of the delta time (BLAIR vs. human) (Figure 5a). In terms of seconds, the majority of data points are clustered around zero, which means that the Blair and human measurements are typically very similar. However, there is a small tail of data points that extends to the right, which means that there are some cases where the BLAIR and human measurements are significantly different. In particular, the BLAIR approach is faster than the human approach since the delta time parameter is computed by subtracting the clinical (human) detection time from the BLAIR detection time. In particular, the BLAIR software is able to predict the bleeding 3 (IQR: 2) seconds in advance.

Then, to evaluate the BLAIR’s ability to identify bleeding with respect to the “gold standard” of human evaluation, the MCA approach was applied, and a plot of the MCA dimension was performed (Figure 5b). The variable “outcome” (concordance between humans and AI in bleeding detection) has very poor significance. This result might suggest that there is no significant difference between the outcomes provided by BLAIR and clinical (human) detection of bleeding.

A similar result can be observed in the plot of the individuals provided by the MCA model (Figure 5c). This plot shows that each individual provides its own behavior and that the different patients can be clustered into different groups according to the values of all the categorical features, with the exception of the “outcome” variable. This result might suggest, once again, that no evidence is observed in differentiating the performance of BLAIR and clinical (human) in identifying bleeding conditions.

## 4. Discussion

Herein, we present a groundbreaking application of AI through specially developed software named BLAIR. This software has the remarkable ability to predict bleeding occurrences during robotic prostatectomy procedures and promptly alert the surgeon.

Our research followed the direction indicated by Rassweiler’s work in 2017 [18], which highlighted the potential of AI in surgery to significantly reduce the death rate associated with complications arising from surgical surroundings, instruments, and procedures.

Indeed, the incorporation of AI in surgery offers substantial advantages over traditional conventional methods [19]. It leads to faster patient recovery times, successful treatment, reduced pain, less bleeding, and a lower risk of infection. Additionally, AI in medicine empowers surgeons with unprecedented control and precision in minimally invasive procedures [20].

Preliminary experiences with AI in robotic surgery have already been presented, including the adoption of CNN for segmenting lung, bladder, and breast cancer types from imaging, as well as navigation assistance in endoscopic pancreatic and biliary procedures [21].

For intra-operative guidance, AI has proven invaluable by providing enhanced visualization and localization during surgery. For instance, 3D prostate shapes have been generated from multiple 2D ultrasound images [22], and a similar approach has been applied to create 3D shapes of abdominal aortic aneurysms using two 2D fluoroscopic images [23]. Furthermore, depth estimation using camera motion and 3D structural environment mapping have been successfully integrated with AI algorithms. Finally, AI technology was integrated with 3D augmented reality (AR) image-guided surgery, allowing for automatic overlapping of the images in a specific phase of the intervention and guiding selective biopsies intraoperatively [24]. In the future, automatic overlapping during the entire procedure can be achieved.

More recently, supervised ML systems have been employed to analyze surgical video streams for instrument detection, segmentation, and pose estimation [25].

De Backer et al. [26] developed an advanced algorithm using deep learning networks to detect robotic instruments in surgery. Trained on a dataset of 65,927 labeled instruments across 15,100 frames, the algorithm achieves real-time instrument delineation through binary segmentation. The researchers integrated their application with 3D models and augmented reality (AR) images, connecting it to a dedicated laptop that combines AR video with real-time instrument detection data. This creates an AR video displaying instrument detection information on the robotic console via an Intuitive TilePro input.

Marullo et al. [13] introduced a multi-task CNN, an architecture able to simultaneously learn multiple tasks. In the case study under consideration, the model was able to perform simultaneous tool detection using semantic segmentation and event detection, specifically bleeding identification, during laparoscopic surgery. The majority of the time, indeed, active bleeding starts when one of the instruments closes or comes into contact with anatomical tissues like the prostate. On the other hand, when the suction device begins to remove the blood from the surgical field, there is a decrease in accumulated bleeding. It was interesting that the network correctly identified the decrease in blood accumulation in the operating scene during the test on the videos, as the percentage fell while the laparoscopic aspirator was eliminating the blood accumulation. The fact that the features recovered from the shared backbone for tool segmentation also proved valuable for event detection suggests that the tasks to be solved simultaneously were chosen appropriately.

In the current days, where more and more young doctors have access to robotics [27], and parallelly, the topic of identification of intraoperative adverse events (iAE) has gained new awareness [28], the adoption of new technologies, such as AI, for intraoperative bleeding detection perfectly fits this scenario. Furthermore, AI-based systems like the proposed one can be applied during surgical training to improve both the surgeon’s performance and the patient’s safety.

Considering its intrinsic exploratory nature, our study is not devoid of limitations.

From a technical standpoint, future studies will concentrate on two factors in order to overcome current limitations. On the one hand, the network’s design needs to be expanded to take into account the temporal information of data gleaned from a series of images within a video instead of considering videos as independent frame-by-frame sequences of data, aiming to increase the accuracy and dependability of the event detection branch. In order to implement a neural network architecture taking into account the temporal pattern in data sequences, a different data source should be provided, namely videos instead of images. This shift may lead to issues in terms of computational power, which is much higher when dealing with video sequences. Nonetheless, there are several benefits to using temporal information; in fact, it allows physicians to better track the bleeding, identify its source, and distinguish between active and passive bleeding. Moreover, temporal information may further improve robustness by integrating data representing unexpected changes in the surgical field into the training dataset.

Additionally, in order to improve prediction, the semantic segmentation task should distinguish between different tools, increasing the number of classes identified by the network, and the bleeding source should be localized to expand its potential clinical benefits when the human eye is unable to detect bleeding immediately. This could be achieved by adding a new branch to the proposed architecture, leveraging on the potentialities of the multi-task learning approach, which allows for simultaneous performance of different classifications, or integrating the semantic segmentation task with blood tracking functionality, blending the information related to the blood accumulation and the identification of the involved region of interest [29] for a full comprehension of the critical event. Moreover, the number of classes could also be increased to better describe the bleeding event, which could be spontaneous or non-spontaneous. In particular, it will be necessary to increase the number of classes from 2 (blood accumulation/no blood accumulation) to 3 (spontaneous bleeding/non-spontaneous bleeding/no bleeding). To this end, the labeling of the images should be further refined to locate other features, including the tip of the cutting instrument and any other regions of interest (e.g., bleeding source).

On the other hand, a dataset with more samples and increased variation might help to enhance prediction accuracy by providing the model with different kinds of data, for instance, RGB and depth videos, following a multimodal approach. In fact, neural networks can generalize the classification performance of a given task to datasets different from those used for training. The training must be performed on sufficiently heterogeneous and variable datasets to allow the neural network to discern between the different anatomical structures displayed within the frames and to perform the event detection phase in a punctual and patient-specific manner, also in relation to anatomical variations among patients. Moreover, generalization is core to handling different boundary conditions, which are not uncommon when dealing with different datasets. Concerning lighting, differences between frames are managed by implementing a preprocessing step involving histogram equalization to enhance image contrast.

From a technological perspective, the adoption of a 3D camera, namely a camera capable of detecting depth, could provide a different source of information and, consequently, allow us to adopt a multimodal approach. In this sense, stereoscopy (passive or active) can provide state-of-the-art results in terms of resolution; nonetheless, the presence of two different points of view could be challenging. For this reason, different 3D acquisition technologies could be considered, such as structured light and time-of-flight, which allow positioning the RGB and the depth sensor very close to each other (ideally in the same exact position), diminishing the probability of occlusions and making the overall size of the acquisition system smaller and more easily placeable in the surgical field.

From a clinical perspective, our BLAIR system was developed based on videos of surgeries performed by experienced surgeons. Consequently, the recorded bleeding events may appear less severe compared to those occurring during surgeries performed by novice surgeons. Additionally, in the event of an unforeseen and abrupt bleeding occurrence that has not been previously recorded by the software, it may encounter challenges in recognizing and responding to it appropriately.

Finally, we would like to underscore the significance of ethical considerations, which are fundamental when AI is employed in healthcare. As of now, this system is not intended to replace the surgeon but has been developed solely as an assistant. Therefore, in the event of software failure, the surgeon retains accountability for the surgical outcomes. In the subsequent phases of software development, these aspects will also be addressed, encompassing not only responsibility but also transparency and an explanation of BLAIR’s decisions.

In the future, the same technology could be used for the identification and recognition of some crucial steps of the intervention, guiding the surgeon during the incision maneuvers. Furthermore, this system should be tested by young surgeons with the aim of verifying the real clinical benefit of the BLAIR software application: the possibility to predict and alert the surgeon of a risk of bleeding can effectively lead to a reduced EBL after the intervention, improving patient safety.

Moreover, this AI-based software, which is able to recognize the different elements in the operative field, will represent the basis for future image-guided augmented reality surgery by using 3D models of the patient’s anatomy.

## 5. Conclusions

Our BLAIR software was able to correctly predict the bleeding occurrence during RARP with more than 90% accuracy. This experience represents how the application of AI can potentially assist the surgeon during the intervention. In the current technology-driven surgery era, AI will be a fundamental player in improving the safety of the intervention, especially at the beginning of the learning curve.

## Figures and Tables

**Table 1:** Patients perioperative characteristics (BMI: body max index; EBL: estimated blood losses; GS: Gleason Score).

Patient ID	Age at Time of Surgery	BMI	LND	Operative Time	EBL	Nerve Sparing	Pathological GS	pT	Prostate Volume
*#1*	69	27	YES	119	350	Full	3 + 4	pT3a	30
*#2*	67	33	YES	124	400	NO	4 + 5	pT3a	38
*#3*	71	31	YES	127	500	NO	3 + 4	pT2c	55
*#4*	71	27	YES	134	300	Partial	3 + 4	pT2c	118
*#5*	75	29	YES	128	400	NO	4 + 4	pT3a	32
*#6*	52	24	YES	149	400	Full	3 + 4	pT3a	15
*#7*	65	24	YES	114	300	Partial	3 + 4	pT2c	68
*#8*	69	23	YES	175	500	NO	4 + 5	pT3b	35
*#9*	62	27	YES	132	350	Partial	4 + 3	pT2c	38
*#10*	70	28	YES	135	400	Partial	3 + 4	pT2c	59

## Data Availability

The data presented in this study are available on request from the corresponding author.
